# Supplementary material for: Examining self-employment policies for persons with disabilities in South Africa: Perspectives from policy actors
Source: PLoS One. 2025 Sep 4;20(9):e0331576. doi: 10.1371/journal.pone.0331576 (PMC12410715; doi:10.1371/journal.pone.0331576)
Supplement: Attachment — Submitted filename: Response to Reviewers letter policy reviewed.docx [file pone.0331576.s002.docx]

**RESPONSE TO REVIEWERS (AUGUST 2025)**

- Kindly request that the reviewers provide explicit and constructive feedback, preferably directly in the manuscript.

| **Comment from journal** | **Authors’ response** |
| --- | --- |
| **When submitting your revision, we need you to address these additional requirements.** | |
| 1. Please ensure that your manuscript meets PLOS ONE's style requirements, including those for file naming. | Done |
| 2. In the online submission form, you indicated that [Data can be accessed by contacting the corresponding author].  All PLOS journals now require all data underlying the findings described in their manuscript to be freely available to other researchers, either 1. In a public repository, 2. Within the manuscript itself, or 3. Uploaded as supplementary information. | The data availability statement was updated as follows:  “All raw data files are available from Open Science Framework (OSF). The full reference is as follows: Monareng LL. PhD Raw Data. Data collection from 2023 to 2024 [Internet]. OSF; 2025. Available from: <https://osf.io/pdfks/>” |
| 3. If the reviewer comments include a recommendation to cite specific previously published works, please review and evaluate these publications to determine whether they are relevant and should be cited. There is no requirement to cite these works unless the editor has indicated otherwise. | Not applicable |
| **Reviewer #1:** | |
| The introduction is too lengthy and sometimes repetitive. It details relevant local international policy frameworks and conventions, but it does not adequately include a critical literature review. For this reason, the study does not appear to be grounded in the existing body of knowledge. Moving ahead, the introduction could be more concisely written. To strengthen, it needs to include more academic literature. | As stated throughout the manuscript, academic literature on self-employment for persons with disabilities is lacking, let alone specific policies in this field. As such, we deemed it necessary to provide a thorough background on this subject to help the reader to follow.  Specifically, the reviewer’s concerns have been addressed as follows:  -We introduced subheadings to assist with flow and removed some parts of the introduction  -The introduction section was reviewed for thoroughness and flow. The content was strengthened by aligning it with literature in the discussion section, as recommended by the reviewer: “The authors may revise the introduction in line with the discussion that includes many sources, making both the sections consistent.”  -Repetitions were identified and removed. |
| The study meets the applicable ethical standards as the authors obtained ethical approval from an institutional ethics committee. | Noted |
| The following sentence requires correction and revision for clarity: “For instance, the context (environment) or contextual factors such as politics affect the processes followed when formulating content (policies) and outcomes thereafter.” (Lines 81-82). In addition, the word ‘capacitated’ (lines 403-404) reads a bit odd. The authors may want to use a more suitable words such as ‘built capacities’. | Lines 81-82: Revised and extended as follows “For instance, the environment (context), including politics affect the processes followed when formulating policies (content) and outcomes thereafter. This suggests that a democratically elected political party runs a country’s affairs, including leading the formulation and implementation of relevant policies through a lengthy consultative process with the country’s citizens.”  ‘capacitated’ (lines 403-404): Inserted the word ‘trained’ next to the word ‘capacitated’. |
| The methods section is detailed and indicates that the research was conducted systematically. | Noted |
| The study has generated important and insightful findings, as reported in the findings sections. There are quite a few quotations presented as evidence for each sub-theme. However, the is a limited analysis and interpretation of the findings. Adding more and deeper analysis could strengthen this section. The findings section lacks flow due to the use of too many quotations in the same place without analysis. | Limited analysis and interpretation  In line with the objectives of this research, we reported the findings using themes and supporting quotes to capture the collective contributions of the participants. As such, interpretation occurred only under the discussion section, where the findings were integrated with existing literature.  Lacks flow due to the use of too many quotations  -Quotes were reduced per subtheme, with only the most crucial ones retained.  -In some cases, quotes were reorganised, refined and shortened  -However, in certain subthemes, up to 5 quotes were deemed necessary and left unchanged, such as the list of inclusive policies under ‘3.2.1.2. Inclusive legal frameworks...’, which captures the breadth of the various policies suggested by the participants – emphasising their point that the country does not need to add more laws but rather focus on implementation or tweaking what is in place. |
| Compared to the introduction, the discussion section is stronger. The authors may revise the introduction in line with the discussion that includes many sources, making both the sections consistent. | No major revisions made in discussion. Flow was enhanced instead. See revisions made in the introduction section to align with discussion. |
| The reference list needs some revisions and needs to be formatted consistently. For example, the first reference is in all capitals, whereas the rest are not. | Revised |
| While revising your submission, please upload your figure files to the Preflight Analysis and Conversion Engine (PACE) digital diagnostic tool, [https://pacev2.apexcovantage.com/](https://url.za.m.mimecastprotect.com/s/xyqmCy8k2RtrNoVBxhxHOix5fPB?domain=track.editorialmanager.com). PACE helps ensure that figures meet PLOS requirements. To use PACE, you must first register as a user. Registration is free. Then, login and navigate to the UPLOAD tab, where you will find detailed instructions on how to use the tool. If you encounter any issues or have any questions when using PACE, please email PLOS at [figures@plos.org](mailto:figures@plos.org). Please note that Supporting Information files do not need this step. | Done |
